# Multivariate Imaging for Fast Evaluation of In Situ Dark Field Microscopy Hyperspectral Data

**DOI:** 10.3390/molecules27165146

**Published:** 2022-08-12

**Authors:** Sabrina Diehn, Helmut Schlaad, Janina Kneipp

**Affiliations:** 1 Department of Chemistry, Humboldt-Universität zu Berlin, Brook-Taylor-Str. 2, 12489 Berlin, Germany; 2 Institute of Chemistry, University of Potsdam, Karl-Liebknecht-Str. 24-25, 14476 Potsdam, Germany

**Keywords:** localized surface plasmon resonances, gold nanoparticles, silver nanoparticles, dark field microscopy, acrylamide, hyperspectral imaging, non-negative matrix factorization, surface-enhanced Raman scattering (SERS), surface-enhanced hyper Raman scattering (SEHRS)

## Abstract

Dark field scattering microscopy can create large hyperspectral data sets that contain a wealth of information on the properties and the molecular environment of noble metal nanoparticles. For a quick screening of samples of microscopic dimensions that contain many different types of plasmonic nanostructures, we propose a multivariate analysis of data sets of thousands to several hundreds of thousands of scattering spectra. By using non-negative matrix factorization for decomposing the spectra, components are identified that represent individual plasmon resonances and relative contributions of these resonances to particular microscopic focal volumes in the mapping data sets. Using data from silver and gold nanoparticles in the presence of different molecules, including gold nanoparticle-protein agglomerates or silver nanoparticles forming aggregates in the presence of acrylamide, plasmonic properties are observed that differ from those of the original nanoparticles. For the case of acrylamide, we show that the plasmon resonances of the silver nanoparticles are ideally suited to support surface enhanced Raman scattering (SERS) and the two-photon excited process of surface enhanced hyper Raman scattering (SEHRS). Both vibrational tools give complementary information on the in situ formed polyacrylamide and the molecular composition at the nanoparticle surface.

## 1. Introduction

The strong elastic scattering of light at visible wavelengths due to localized surface plasmon resonances (LSPR) is the basis of a vast range of applications that employ the high sensitivity of the LSPR of noble metal nanoparticles. The LSPR of a single gold or silver nanoparticle gives detailed information about its size, shape, and interaction with other nanoparticles [1,2,3,4,5,6], and about changes that occur at the particle surface, including the adsorption of atoms or molecules [7,8,9,10], down to few-molecule level [11]. Moreover, small changes in surface charge modify the scattering spectrum [12], enabling, e.g., monitoring of redox reactions on individual nanoparticles [13]. Dark field optical microscopy visualizes the scattering of plasmonic nanoparticle arrays and ensembles [14]. It has become an important tool for fast probing of the resonances of new plasmonic substrates, e.g., for surface-enhanced Raman scattering (SERS) [15,16,17], tip-enhanced Raman scattering (TERS) [18], plasmon-induced magnetic resonance [19], second-harmonic generation (SHG) [20], and hot electron generation [21].

While machine learning tools [22,23,24] and automated correction [25] can be used to process images obtained in dark field microscopy directly, it is desirable to utilize them for assessment of the full scattering spectra, so that information on different plasmon modes and their sensitivity to changes in environment can be obtained from the full hyperspectral data [26,27,28,29]. Imaging of scattering intensity at particular wavelength [26] and introducing a color code to represent the wavelength of maximum scattering [30] resemble the “chemical mapping” univariate in other analytical applications, such as imaging mass spectrometry, Raman or FTIR microspectroscopy. In samples in which individual nanostructures are not separated from one another further than the diffraction limited spatial resolution, probing by hyperspectral dark field microscopy leads to massive superposition of resonances from many different plasmon modes in all individual nanostructures contained in one focal volume. Assemblies of plasmonic nanoaggregates up to the micron size range are common in different molecular environments that can be studied by dark field microscopy. They include nanoparticle accumulations in living cells, refs. [26,29] substrates in plasmonic catalysis [28,31], or plasmonic nanoparticles in composite materials, such as polymers [32]. The scattering spectra of different microscopic pixels in a hyperspectral map can vary greatly, depending on their composition with respect to nanoparticle aggregate size, shape, molecular environment, or orientation.

Here, we decompose the hyperspectral information in dark field scattering microscopy data with the aim of mapping the dominating contributions of distinct plasmon resonances at specific microscopic positions in a sample. We use hierarchical cluster analysis and non-negative matrix factorization (NMF) on data sets of many thousands of spectra that were acquired from gold and silver nanoparticles immobilized in the presence of a protein and acrylamide, respectively, as simplified molecular environments. As will be discussed, the specific types of nanoparticle agglomerates that are forming in the samples enable sensitive probing of the molecular environment by one- and two-photon excited SERS.

## 2. Results and Discussion

**Figure 1:**
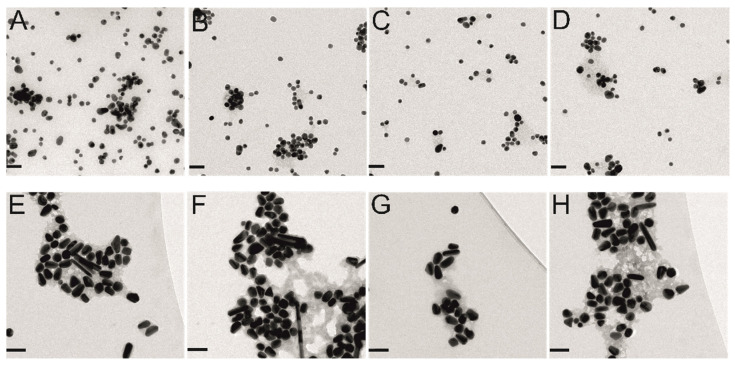
Transmission electron micrographs of the nanoparticles used in this study. (**A**–**D**) Citrate stabilized gold nanoparticles, scale bars: 50 nm. (**E**–**H**) Citrate stabilized silver nanoparticles, scale bars: 200 nm.

Gold and silver nanoparticles, both stabilized in aqueous solution and by citrate ions were drop cast on microscope slides. Transmission electron micrographs of the nanoparticles are shown in Figure 1. Both types of nanoparticles have been used in applications of SERS in different context, the gold nanoparticles as SERS nanoprobes in cells, with and without specific molecular functionalization, refs. [33,34] and the silver nanoparticles as substrates for SERS and surface-enhanced hyper Raman scattering (SEHRS) studies of different organic molecules, for example in the context of plasmon-supported reactions [35,36]. The size of the gold nanoparticles (Figure 1A–D) was determined to be about 14 nm, refs. [33,34] that of the silver nanoparticles (Figure 1E–H) to about 70 nm, with many of the latter showing non-spherical shape.

### 2.1. Exploring Large Dark Field Scattering Data Sets of Silver Nanoparticles

**Figure 2:**
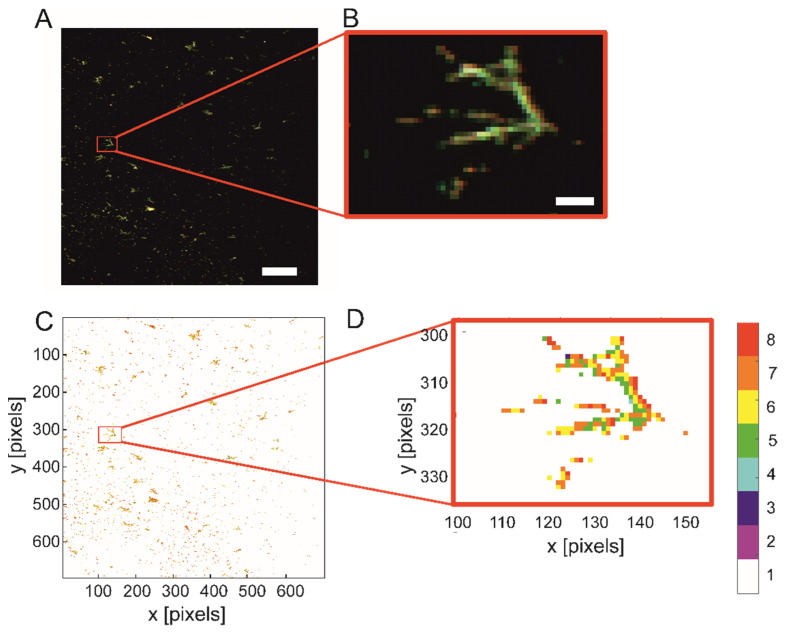
Dark field scattering data set of silver nanoparticles. (**A**) RGB image based on scattering intensity at 660 nm, 540 nm, and 470 nm. Scale bar: 20 µm. (**B**) Magnification of a region of interest. Scale bar: 2 µm. (**C**,**D**) Visualization of the result of a classification analysis generating eight clusters that represent scattering signals at different spectral regions: Cluster 1, white, no signal, Cluster 2, magenta, 400–450 nm, Cluster 3, blue, 450–490 nm, Cluster 4, cyan, 490–520 nm, Cluster 5, green, 520–560 nm, Cluster 6, yellow, 560–590 nm, Cluster 7, orange, 590–635 nm, Cluster 8, red, >635 nm.

Figure 2A shows an RGB image of the silver nanoparticles, generated by using the unit counts at the red, green, and blue wavelength of 660 nm, 540 nm, and 470 nm, respectively. For each wavelength, a map with 696 × 696 pixels was created. The three maps were afterwards normalized using a min/max normalization and combined to a “true color” image. The scattering of the nanoparticle aggregates is shown in lighter colors, and many pixels where no nanoparticles are present are colored black in the image. A magnification of some areas (Figure 2B) reveals agglomeration and assembly into lines, some with dendritic structures that suggest the typical self-similar assemblies discussed frequently in the past [37,38,39].

The images shown in Figure 2A,C are produced from 484,416 spectra. For a quick exploratory analysis, including hierarchical cluster analysis or principal component analysis, it is desirable to drastically reduce the size of the data set without losing information. In the example of the silver nanoparticles shown in Figure 2, a fast and simple classification approach was applied, that assigned all of the 484,416 spectra into seven classes, with respect to their maximum scattering wavelength, as well as one class of spectra that can be excluded, as they come from the “dark” regions that do not show any signal in the RGB image (Figure 2A,B). In principle, the pixels in Cluster 1 are not relevant for describing the optical properties of the sample and can be excluded from the data set (Figure 2C,D). If a spectrum shows no bands determined by a pre-set threshold, the spectrum is assigned to the group of data points with no signal (here Cluster 1). In the map, each spectrum is represented with a false color pixel that is assigned to each spectral class. Most of the data points in the map can be excluded using this algorithm, leaving 31,670 of 484,416 spectra for a detailed analysis in this sample. In the magnified structure in Figure 2D, scattering maxima due to plasmon resonances are mostly located in the range of 520–700 nm.

Automatic selection of spectra with actual information based on a signal threshold is an efficient way to reduce computation time in an analysis. Similarly, large data sets can be divided into regions of interest (ROI), as proposed in previous studies [27,29]. As examples, maps of ~10,000 spectra can be processed easily without prior extraction of spectra.

**Scheme 1:**
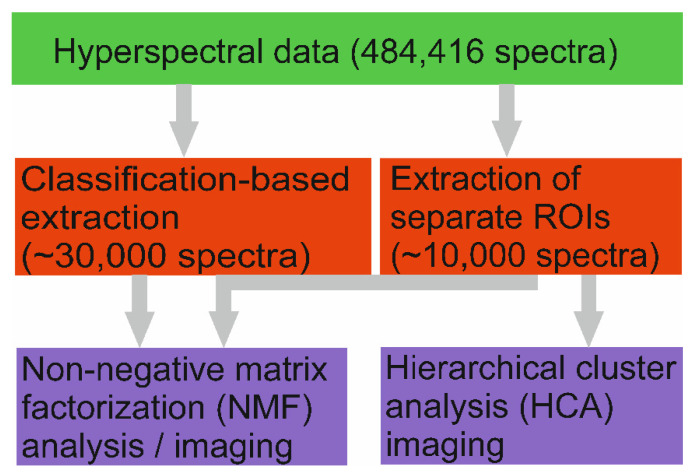
Different possibilities used for multivariate screening of the hyperspectral data sets.

To screen subsets of data, we use hierarchical cluster analysis (HCA) as an exploratory analysis tool in order to obtain further information about the predominating plasmon resonances at different positions in the samples. HCA can reveal more information about similarities of the spectra, considering the full spectral range that can have several plasmon resonances. Moreover, as will be discussed later, non-negative matrix factorization (NMF) will be used to decompose each individual spectrum into different contributions of a set of NMF components. Scheme 1 summarizes the data analysis proposed here.

**Figure 3:**
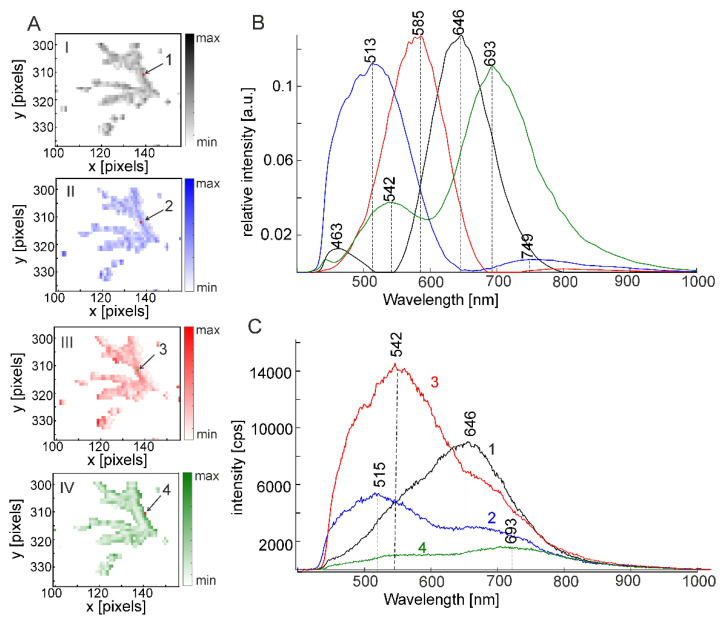
(**A**) NMF maps corresponding to the four pure components of the magnified region of the dark field image of silver nanoparticles presented in Figure 2. The NMF maps visualize the relative contribution of each of the four pure components. Therefore, each pixel has an intensity value between 0 and 1, and the sum of all contributions to the same pixel in each map equals 1. (**B**) The four NMF components. The colors of the component spectra correspond to those of the NMF maps in (**A**). (**C**) Extracted spectra from the marked spots in (**A**).

The results of the NMF analysis of the data set acquired from the silver nanoparticles discussed above are shown in Figure 3.

The decomposition was executed for different numbers of components (data not shown), and the amount of four components was found to be optimal, as it led to well-separated, relatively narrow bands in the component spectra. Figure 3A shows the NMF maps of the four components for the whole image of the silver nanoparticle sample. The component spectra, plotted in Figure 3B, contribute differently at different positions in the map. One pure component represents part of the resulting spectrum in a microscopic volume (pixel), e.g., one or two particular plasmon resonances, so a detailed description of each spectrum also needs to take into account the other respective components as well. Figure 3C shows four individual spectra selected in each NMF map, where a high portion of the corresponding pure component is present (from I, II, III, and IV in Figure 3A). They were extracted from the image and can be compared to the pure component spectra of each of the maps (compare Figure 3C with Figure 3B). As an example, one of the components (Figure 3B, black trace) of the NMF map has maxima at 463 nm and at 646 nm. The original spectrum from a pixel in the corresponding map (Figure 3A, black map, I) with high contribution of that component (Figure 3B) shows maxima at the same positions, with the low wavelength contribution by the typical dipolar plasmon mode of spherical silver nanoparticles around 460 nm here as shoulder. The other extracted spectra 2, 3, and 4 (Figure 3A,C blue, red, and green) also show similar maxima as the respective component spectra (Figure 3B,C blue, red, and green, respectively).

The example in Figure 3 illustrates that NMF can really serve as a explorative analysis tool, in order to gain information about the most possible scattering maxima and their contribution to the spectra that are obtained from different regions in the sample. The maps of the individual components (Figure 3A) indicate different scattering maxima in the spectra at the outer border of the microscopic structure, than in the spectra in the middle parts. We also performed HCA of the data set of the silver nanoparticles, where we also found that the spectra are sorted into clusters with respect to the shape of the microscopic agglomerates (data not shown here). The application of HCA to the dark field scattering data sets is further discussed for the example of different types of gold nanoaggregates below.

### 2.2. Agglomerates of Gold Nanoparticles with and without Protein

**Figure 4:**
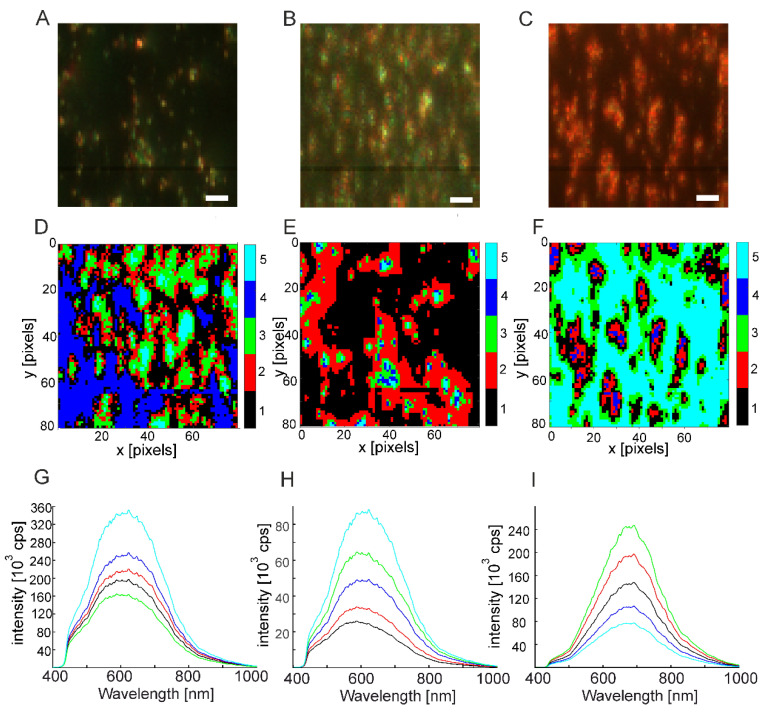
RGB images obtained from data sets of (**A**) gold nanoparticles, (**B**) gold nanoaggregates prepared with sodium chloride and (**C**) gold-BSA agglomerates. Each mapping data set comprises 6399 spectra. (**D**–**F**) Respective hyperspectral maps from the result of hierarchical cluster analyses (HCA) applied to the data sets without pre-processing. Mapping was based on five spectral classes; each assigned a different color. (**G**–**I**) Average spectra calculated from the different classes in each data set; colors correspond to pixel colors in D, E, and F, respectively. Scale bars: 2 µm.

Gold nanoparticles are often used for optical probing of biological samples, by dark field microscopy as well as by SERS. Understanding the formation of aggregates in the presence of proteins is of particular interest for such applications, since the nanoparticles are usually surrounded by a biomolecular corona that is formed by different proteins contained in the cell, the extracellular environment, or a cell culture medium. Small gold nanoparticles of ~14 nm diameter, used for SERS applications were shown previously to form agglomerates in protein solution, specifically in the presence of typical serum proteins such as bovine serum albumin (BSA) [34]. Three different samples of these nanoparticles were studied by dark field microspectroscopy here: (i) As-prepared citrate stabilized gold nanoparticles (Figure 4A,D,G), (ii) aggregates of the same gold nanoparticles formed by adding sodium chloride in a final concentration of 100 mM (Figure 4B,E,H), and (iii) agglomerates of the nanoparticles formed in the presence of 15 µM BSA as discussed in previous work [40] (Figure 4C,F,I). The maps here (Figure 4A–C) are ROI of large maps and have a size of 79 pixels × 81 pixels (6399 spectra) each. In Figure 4 we show three RGB images (Figure 4A–C) and maps based on the assignment of the spectra to different classes as a result of a hierarchical cluster analysis (HCA) (Figure 4D–F) of the three samples. HCA sorts similar spectra into clusters so that the corresponding pixels in the map can be marked with a particular color for the same cluster. The RGB images (Figure 4A–C) differ from that of the gold nanoparticle-protein agglomerates showing more orange and red colors (Figure 4C) than the two other maps. The HCA maps (Figure 4D–F) were generated without any pre-processing of the spectra and therefore also identify regions of high and low signal in the microscopic sample. All three HCA images (Figure 4D–F) indicate similarities of spectra in similar regions of the microscopic aggregates. High scattering signals are found for the inner parts of the clusters in the samples prepared without BSA (Figure 4G,H), while high absolute intensities are found in the outer zones of the agglomerates in the sample prepared with BSA (Figure 4F,I, green pixels and green trace). Microscopic structures can be identified, where the spectra are changing from the inner part to the outer part of the larger accumulations of the gold nanoparticles.

The averaged spectra from each of the classes (Figure 4G–I) show maxima at different positions in the three samples and also between the different classes within each map. In the aggregates formed by adding sodium chloride (Figure 4E,H), a shift is observed from the regions of lower scattering intensity (black), mainly caused by individual nanoparticles and/or small aggregates, and those from the center of the microstructures (blue traces), that have contributions from more red-shifted resonances. In agreement with the RGB image, all averaged spectra in the sample prepared with BSA show a red shifted maximum compared to those in Figure 4G,H and also lack a pronounced shoulder at low wavelength.

**Figure 5:**
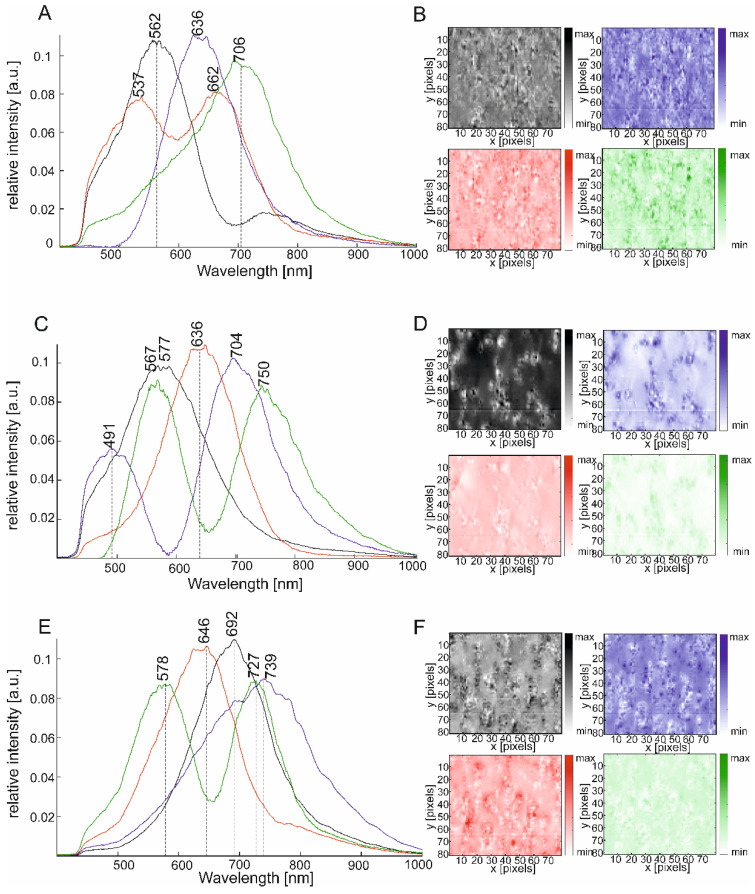
Results of the NMF decomposition of data sets of (**A**,**B**) gold nanoparticles, (**C**,**D**) gold nanoaggregates generated by adding sodium chloride, and (**E**,**F**) agglomerates of gold nanoparticles and BSA. (**A**,**C**,**E**) Four components that were used to construct four maps in (**B**,**D**,**F**) in corresponding color for each of the samples. The maps indicate the relative contributions of the respective component in each of the data points.

Since most information on the individual spectra is lost during averaging of the spectra after the cluster analysis, an analysis of the individual pixel spectra was performed using NMF. Moreover, here, a decomposition into four components proved optimal to resolve different resonances while enabling comparison of similar bands between the different samples. Figure 5 shows the results of the decomposition by NMF as maps that are based on the relative contribution of each of the four components to each pixel (Figure 5A,C,E), resulting in four images for each of the three samples (Figure 5B,D,F, matching colors).

In all samples, the maps show high contributions by some of the components that are complementary and also indicate that plasmonic properties of the clusters change from the inner to the outer parts of the microscopic structures. As an example, the black and blue or green maps of Figure 5D highlight different resonance wavelengths, as evidenced by the respective pure component spectra in Figure 5C. The component shown in black (Figure 5C) indicates a resonance with a maximum at 577 nm, while the component displayed in blue (Figure 5C) shows a strong contribution at 704 nm, which typically present in large nanoaggregates that must be characteristic of the centers of the microscopic nanoparticle formations here (Figure 5D, blue map). The latter spectra were also sorted to the cluster that is shown in light blue in Figure 4D,H.

Although the data from all three different samples yield pure component spectra that range in signal from ~500 nm to ~1000 nm (compare the traces in Figure 5A,C,E), the shape and positions of the maxima differ a lot. While the samples that do not contain BSA yield components with a pronounced contribution around 490–500 nm (Figure 5A, red trace, 5C, blue trace), such a resonance is absent from the data of the gold-BSA agglomerates (Figure 5E). A component with a maximum ~560 nm is missing in the gold-BSA sample as well. Both data sets from pre-aggregated samples yield a pure component with a maximum at ~577 nm (Figure 5C, black trace, Figure 5E, green trace) and a maximum ~750 nm (Figure 5C, green trace, Figure 5E, blue trace), suggesting the presence of aggregates with characteristic resonances that cannot be found in the sample of the pure gold nanoparticles. In the gold-BSA agglomerates (Figure 5E), the band is very wide, with higher contribution in the longer wavelengths, in agreement with the observed red shift of the cluster average spectra found in Figure 4I.

### 2.3. Silver Nanoparticles in Polymerizing Samples

**Figure 6:**
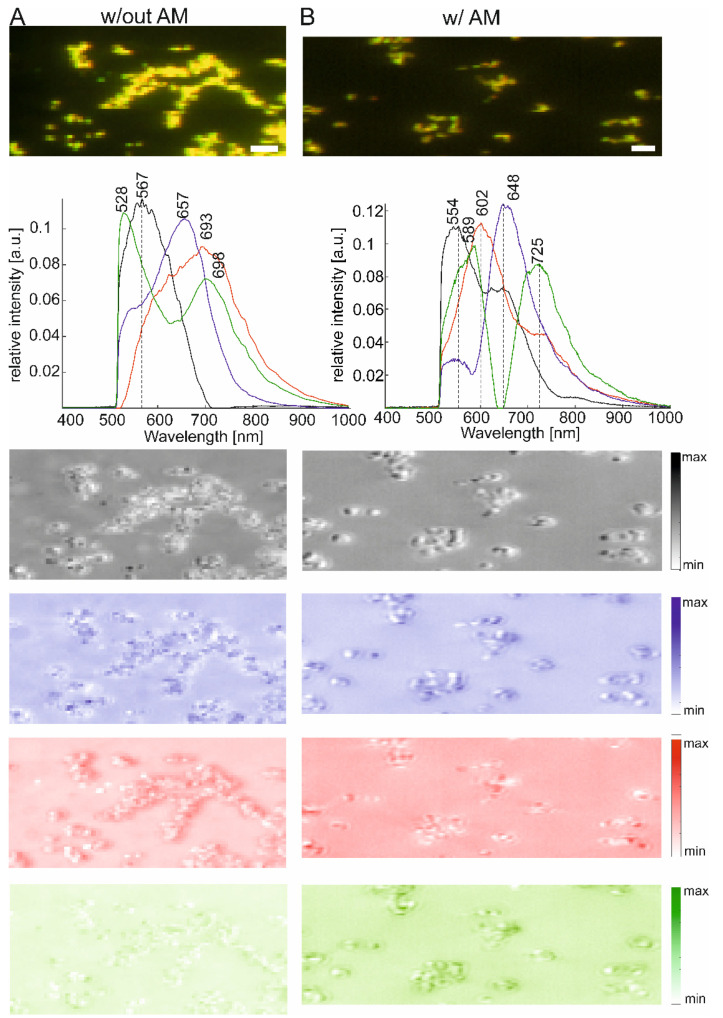
RGB image and NMF components of a dark field scattering data set of silver nanoparticles (**A**) in the presence of MgSO_4_ and (**B**) in the presence of MgSO_4_ and acrylamide. The sizes of the maps are (**A**) 105 × 49 spectra and (**B**) 176 × 59 spectra. Scale bars: 2 µm.

Silver nanoparticles were studied in the presence of Mg^2+^, previously discussed to enhance surface enhanced Raman scattering (SERS), surface enhanced hyper Raman scattering (SEHRS), as well as plasmonic chemistry [41,42,43,44]. Figure 6 shows two regions of interest in dark field scattering maps of two samples, one containing only MgSO_4_, the other MgSO_4_ and acrylamide. The RGB images indicate the formation of aggregated structures (Figure 6A,B, enlarged insets). The nanoparticles in the sample prepared without acrylamide form large, microscopic structures that often have dendritic shape, similar to the ones shown in Figure 2 (Figure 6A). Several organic components have been described to promote the formation of dendritic structures of silver nanoparticles in the past [45,46].

The decomposition into four NMF components reveals a component with a very wide maximum, extending from ~695 nm to ~760 nm, and the sample containing acrylamide shows a strong contribution from a component that has a scattering maximum at 725 nm that appears narrower (Figure 6B, green trace). Comparing both samples with the sample discussed in Figure 3, the low wavelength component at 513 nm (Figure 3B) is replaced by a maximum at 528 nm (Figure 6A, green trace), also supporting previous observations that efficient nanoaggregate formation occurs in the presence of MgSO_4_ [42]. In the sample containing acrylamide, this resonance is missing, instead, components are found at 554 nm and ~600 nm (Figure 6B, black and red trace). Both data sets yield a pure component with a maximum at ~650 nm (blue traces in Figure 6A,B). The corresponding maps yield high contrast and show that the positions where the different components contribute strongly do not overlap.

**Figure 7:**
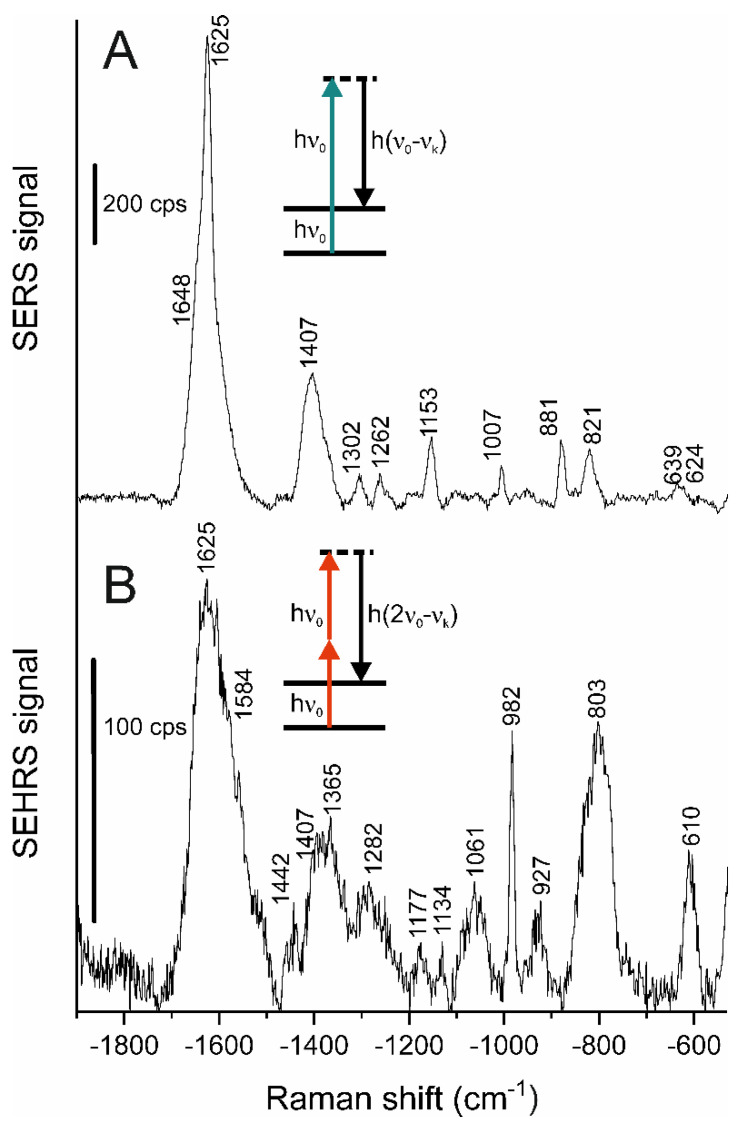
(**A**) SERS and (**B**) SEHRS spectrum of a sample containing silver nanoparticles, MgSO_4_, and 10^−5^ M acrylamide, indicating the formation of polyacrylamide in the acrylamide containing samples. Excitation conditions: (**A**) 532 nm, 1.9 × 10^27^ photons cm^−2^ s^−1^ (**B**) 1064 nm, 3.4 × 10^28^ photons cm^−2^ s^−1^. Acquisition time: (**A**) 1 s, (**B**) 10 s, average of 67 spectra. The insets in (**A**) and (**B**) depict the Raman and the hyper Raman scattering process, respectively.

The component around ~550 nm should be ideal to support optical processes that benefit from plasmon resonances in this spectral region, in particular Raman and hyper Raman scattering (HRS), when Stokes-shifted relative to an excitation wavelength of 532 nm and 1064 nm (for two-photon excited hyper Raman scattering, cf. inset in Figure 7B), respectively. Therefore, these vibrational spectra can provide an additional perspective on the properties of the silver nanoaggregates, by characterizing the molecules that may be involved in the aggregate formation, and their interaction with the nanoparticle surface. Both, spontaneous Raman scattering (SERS) spectra with excitation at 532 nm (inset in Figure 7A), as well as two-photon excited, surface enhanced hyper Raman (SEHRS) spectra with an excitation wavelength of 1064 nm (inset in Figure 7B) will give information on the molecules that are in close proximity to the silver surface [47]. SEHRS is the two-photon analogue of SERS, but follows different selection rules, and is far more sensitive to small changes in surface potential and molecular adsorption [48]. We observed in the microscope the formation of polyacrylamide microscopic structures, of which we obtained SEHRS and SERS spectra. Samples containing no acrylamide did not yield a spectrum.

The SERS spectrum clearly indicates the spectral bands assigned to the polymer (Figure 7A). The data are in agreement with SERS spectra of acrylamide and polyacrylamide, with strong signals from the C-N stretching mode at 1407 cm^−1^ and the polymer chain and side chains at 1153 cm^−1^, 881, and 821 cm^−1^, ref. [49] and the pronounced stretching vibration of the C=O group of polyacrylamide that may also contain some contributions from the C=C stretching of acrylamide at 1625 cm^−1^ [49].

The SEHRS spectrum is excited off-resonance with the acrylamide molecule that is present at 10-micromolar concentration. This is a clear indication of a close proximity of the poly/acrylamide with the surface of the nanoparticles and a formation of efficient hot spots, as is also suggested by the dark field scattering data that indicate aggregate formation. Interestingly, the SEHRS spectrum also provides evidence that the acrylamide monomer must be present at the surface of the nanoparticles as well. This is indicated by several bands, including the vinyl CH_2_ deformation mode at 1282 cm^−1^, the band at 1442 cm^−1^ that is assigned to both the deformation of CH_2_ groups and the C-N stretching in acrylamide, the NH_2_ twisting vibration that is visible at 1134 cm^−1^ (that is distinguished from the skeletal vibration of the polymer at 1177 cm^−1^), and a so far unassigned band at 610 cm^−1^ that was also reported in SERS spectra of acrylamide but not found in polyacrylamide [49,50]. The very pronounced signal at 982 cm^−1^, an HCCH out-of-plane bending mode, has also been assigned to acrylamide, ref. [49] but could also come from polyacrylamide [50]. It has been described as weak in RS and SERS spectra, ref. [49] and its strong enhancement in the SEHRS spectrum could give complementary information to the SERS data regarding orientation of the polymer. In addition to the fact that the probing by SEHRS is even more restricted to the nanoparticle surface than in the SERS data [47], the altered relative intensities of some vibrational modes found here can give valuable complementary information on the polymer structure.

Polymerization reactions, including acrylamide polymerization, ref. [49] in the presence of silver nanoparticles were usually performed using a radical source, refs. [32,51] but here no initiator is present. We suppose that the polymerization is mainly promoted by the presence of the silver nanoparticles, probably by silver ions that we found to help plasmon-catalyzed oxidation reactions at the surface of silver nanoparticles [35]. In addition to providing a source of silver ions, the plasmonic properties of the silver nanoparticles may play an important role in promoting the reaction, particularly since there is no molecular resonance known for the acrylamide molecule itself that could serve in a direct photochemical production as reported by others [52]. We are currently delineating the potential role of the silver nanoparticles in a plasmon-supported polymerization approach, since it could be beneficial for a microscopic in situ synthesis, similar to the laser-direct plasmonic writing that we reported recently [53].

## 3. Materials and Methods

### 3.1. Sample Preparation

Gold(III) chloride trihydrate (HAuCl_4_·3H_2_O, 99.9% trace metals basis) and silver nitrate (99.9999% trace metals basis), bovine serum albumin, and acrylamide were purchased from Sigma-Aldrich. Trisodium citrate dihydrate (99%) was obtained from Th. Geyer, sodium chloride and magnesium sulfate were purchased from J. T. Baker. All solutions were prepared using Milli-Q water (MilliPore purification system).

Silver nanoparticles of a size of ~70 nm were synthesized by citrate reduction as described previously [54]. Gold nanoparticles of a size of ~14 nm were synthesized as described previously [55]. Transmission electron micrographs of the nanoparticles were taken using a Tecnai G2 20 TWIN instrument operating at 200 kV.

The silver colloids were mixed with solutions of 1 mol/L MgSO_4_, 0.1 g/mL, and/or 10^−4^ mol/L acrylamide. The gold colloids were mixed with solutions of 1 mol/L NaCl, or 0.1 g/mL (0.15 mM) bovine serum albumin. The volume ratio was 9:1 (nanoparticles: NaCl or protein) and 8:1:1 (nanoparticles: MgSO_4_: acrylamide). The samples were drop cast on glass slides, dried and sealed under cover glasses. For SERS and SEHRS experiments, the liquid samples were placed in microcontainers.

### 3.2. Dark Field Scattering Microscopy

Dark field images of the nanoparticles were obtained using a microscope (BX51, Olympus, Hamburg, Germany) with a hyperspectral camera (CytoViva, Auburn, AL, USA), and a magnification of 60× (oil immersion, numerical aperture of 1.25). Images of 696 × 696 pixels were collected using an exposure time of 1 s, 0.5 s or 0.25 s. Whole data cubes of 696 × 696 pixels with one scattering spectrum for each pixel (484,416 spectra) were acquired. Regions were defined in ENVI 4.8 software (L3 Harris Geospatial, Boulder, CO, USA). The images were analyzed and visualized using self-written routines in Matlab 2016a (The Mathworks, Inc., Natick, MA, USA).

### 3.3. SERS and SEHRS Experiments

The SERS and SEHRS measurements on the aqueous samples were carried out in microcontainers. The excitation light was focused onto the samples with a microscope objective (NA 0.3). The Raman and hyper Raman scattered light were detected by a liquid nitrogen cooled CCD detector. Spectral resolution was 3–6 cm^−1^, considering the full spectral range. Excitation of the SEHRS spectra was achieved with a 1064 nm mode-locked laser, generating 7 ps pulses at 76 MHz repetition rate. Excitation of SERS spectra at 532 nm was obtained using the second harmonic of the same laser. SEHRS spectra were accumulated for 10 s with an excitation peak photon flux density of 3.4 × 10^28^ photons cm^−2^ s^−1^ (average power 500 mW). SERS spectra acquired for 1 s per spectrum and excited using 1.9 × 10^27^ photons cm^−2^ s^−1^ (average power 16 mW). All spectra were frequency calibrated using a spectrum of toluene. Baseline correction by asymmetric least squares (AsLS) was performed on the SEHRS spectra.

### 3.4. Multivariate Analysis

Hyperspectral data were exported as full data sets or regions of interest from ENVI 4.8 software into MatLab resulting in data cube of a size of 696 pixels × 696 pixels × 472 wavelengths in the case of a full data set. For construction of the RGB images, a 2D intensity map at 660 nm (red), 540 nm (green), and 470 nm (blue) was extracted from the data cube, respectively. The three intensity maps were min-max normalized based on their overall maximum value and subsequently merged into one 696 pixels × 696 pixels × 3 image.

For classification according to spectral color, the 484,416 spectra of the data cube were sorted into eight groups, based on their scattering maxima in eight spectral regions, defined by a signal threshold. The ranges are 400–450 nm, 450–490 nm, 490–520 nm, 520–560 nm, 560–590 nm, 590–635 nm, and above 635 nm. Classification was executed on spectra without further pre-processing.

Hierarchical cluster analysis was carried out on the spectra of defined region of interests using the full spectral range from 400–1000 nm using linkage function of Matlab. Euclidean distance measure and Ward’s algorithm were used for clustering. Cluster maps were based on five spectral classes. Non-negative matrix factorization was carried out using the nnmf function in Matlab. The spectra were taken from the data sets of the full image (696 pixels × 696 pixels) or the regions of interest as indicated in the maps without further pre-processing.

## 4. Conclusions

The results shown here indicate that decomposition of extremely large dark field scattering hyperspectral images of microscopic samples that contain plasmonic nanostructures can be achieved using multivariate approaches, including non-negative matrix factorization. Fast exploration of such large data sets becomes particularly important with the advent of new, fast acquisition methods of dark field hyperspectral data [56]. Although the far-field, diffraction limited probing cannot be used to characterize individual nanostructures or aggregates, different microscopic regions in a sample can be characterized quickly regarding their plasmonic properties and molecular environments. As was discussed for the example of nanoparticles in the presence of different organic molecules, multivariate imaging based on the dark field scattering spectra can provide information on the nanoparticles, while plasmon-supported spectroscopies provide a perspective on the molecular environment. This may prove useful in optical probing in microspectroscopy and other applications, including plasmonic catalysis and in situ synthesis of nanoparticle-polymer composites.

## Data Availability

Original data can be provided upon reasonable request.

## Sample Availability

Samples of the compounds are available from the authors.
